# Enhanced immunogenicity elicited by a novel DNA vaccine encoding the SARS-CoV-2 S1 protein fused to the optimized flagellin of *Salmonella typhimurium* in mice

**DOI:** 10.1128/spectrum.02549-23

**Published:** 2023-11-01

**Authors:** Li Song, Qiaoju Wang, Yaya Wen, Ruimeng Tan, Yaodan Cui, Dan Xiong, Xinan Jiao, Zhiming Pan

**Affiliations:** 1 Jiangsu Key Laboratory of Zoonosis, Yangzhou University, Yangzhou, Jiangsu, China; 2 Jiangsu Co-innovation Center for Prevention and Control of Important Animal Infectious Diseases and Zoonoses, Yangzhou University, Yangzhou, Jiangsu, China; 3 Key Laboratory of Prevention and Control of Biological Hazard Factors (Animal Origin) for Agrifood Safety and Quality, Ministry of Agriculture of China, Yangzhou University, Yangzhou, Jiangsu, China; University of Georgia, Athens, Georgia, USA

**Keywords:** SARS-CoV-2, S1, antibiotic-resistance gene-free vector, DNA vaccine, optimized flagellin adjuvant, immunogenicity

## Abstract

The development of safe and efficient vaccines against coronavirus disease 2019 (COVID-19) is necessary for global public health. The spike protein of severe acute respiratory syndrome coronavirus 2 (SARS-CoV-2) is a major target for the COVID-19 vaccines. Among the available vaccine types, DNA vaccines are a promising alternative to conventional vaccines. Here, we developed a novel DNA vaccine containing the SARS-CoV-2 S1 subunit and an optimized flagellin adjuvant (FliCΔD2D3) from *Salmonella typhimurium*. The S1 gene and S1-*fliC*ΔD2D3 fusion gene were codon optimized and then cloned into the antibiotic-resistance-gene-free vector *asd*-pVAX1, producing *asd*-pVAX1-S1 and *asd*-pVAX1-S1-*fliC*ΔD2D3, respectively. The expression of S1 and S1-FliCΔD2D3 proteins was confirmed after transient expression in HEK293T cells. After *in vitro* characterization of the vaccine candidates, we evaluated the humoral and cellular immune responses induced in BALB/c mice. The DNA vaccine induced a long-lasting humoral response for approximately 4 months, and the elicited serum antibodies efficiently blocked the binding of the SARS-CoV-2 S1 receptor-binding domain to its entry receptor (angiotensin-converting enzyme 2). Moreover, the DNA vaccine induced strong Th1/Th2-mixed immune responses, as demonstrated by the similar levels of S1-specific immunoglobulin (Ig) G1 and IgG2a in the serum and the significantly elevated Th1-type (IFN-γ and TNF-α) and Th2-type (IL-4 and IL-6) cytokine expression from splenic lymphocytes. Importantly, *asd*-pVAX1-S1-*fliC*ΔD2D3 immunization in mice could significantly enhance the levels of immune responses compared to *asd*-pVAX1-S1. This study provides crucial information regarding the selection of a safer DNA vector and a new adjuvant for the vaccine development of SARS-CoV-2 and other infectious diseases.

**IMPORTANCE:**

The development of safe and effective vaccines is needed to control the transmission of coronavirus disease 2019 (COVID-19). Synthetic DNA vaccines represent a promising platform in response to such outbreaks. Here, DNA vaccine candidates were developed using an optimized antibiotic-resistance gene-free asd-pVAX1 vector. An optimized flagellin (FliC) adjuvant was designed by fusion expression to increase the immunogenicity of the S1 antigen. S1 and S1-FliCΔD2D3 proteins were strongly expressed in mammalian cells. The FliCΔD2D3-adjuvanted DNA vaccine induced Th1/Th2-mixed immune responses and high titers of neutralizing antibodies. This study provides crucial information regarding the selection of a safer DNA vector and adjuvant for vaccine development. Our FliCΔD2D3-adjuvanted S1 DNA vaccine is more potent at inducing both humoral and cellular immune responses than S1 alone. This finding provides a new idea for the development of novel DNA vaccines against COVID-19 and could be further applied for the development of other vaccines.

## INTRODUCTION

Since it began in December 2019, the coronavirus disease 2019 (COVID-19) pandemic has caused more than 769 million infections with severe acute respiratory syndrome coronavirus 2 (SARS-CoV-2) and nearly 6.9 million deaths worldwide as of 10 August 2023 (1). Furthermore, the high transmissibility rate of SARS-CoV-2 among humans as well as the emergence of new variants of concern (VOCs) of the virus pose significant obstacles to controlling its spread (2, 3), highlighting the urgent need for the development of safe, effective, and equitably accessible vaccines. In this context, many research institutions need to make rapid progress in developing new vaccines (4). More cutting-edge vaccines, such as virus-like particle, RNA, DNA, and subunit vaccines, are widely studied (5). Among them, DNA vaccines are convenient and rapid in the process of vaccine production owing to their straightforward design, rapid production, low production cost, good temperature stability, and uncomplicated quality control ability (6, 7). Hence, the DNA vaccine platform is suitable for rapid and large-scale manufacturing during infectious disease outbreaks.

Previous studies have reported that DNA vaccines can effectively stimulate humoral and cellular responses against pathogens in challenge models (8, 9). Currently, the DNA vaccine ZycoV-D has been approved for emergency use in India, and 17 DNA vaccine candidates are in preclinical development (10). Most of them are based on the full-length or truncated spike (S) protein of SARS-CoV-2 because the S protein has the ability to activate immune responses. S protein comprises a globular head S1 subunit containing the receptor-binding domain (RBD) and a membrane-proximal S2 subunit (11); S1 can bind to the angiotensin-converting enzyme 2 (ACE2) receptor on the surface of host cells, and S2 is related to the virus-host cell membrane fusion (12). Therefore, neutralizing antibodies (NAbs) against SARS-CoV-2 function mainly by targeting the RBD of the S1 subunit, thereby preventing viral entry into host cells.

Notably, all of these DNA vaccines and vaccine candidates contain antibiotic resistance genes, which can be transmitted into the human microbiome through horizontal gene transfer, thereby increasing the incidence of antibiotic-resistant infections worldwide (13). Consequently, the safety of DNA vaccines is still challenging. In the early stage of vaccine development, our laboratory optimized the commercialized DNA vaccine vector pVAX1 by introducing the *Salmonella* aspartate-beta-semialdehyde dehydrogenase (*asd*) gene into this plasmid, simultaneously destroying the kanamycin resistance gene in pVAX1, to construct the non-antibiotic-resistance gene-containing DNA vaccine vector *asd*-pVAX1. Curtiss et al. first proposed that the use of a balanced lethal system with *asd* as the marker gene could enable screening without the need for antibiotic-resistance genes and thus improve the safety of DNA vaccines while ensuring the stable expression of exogenous antigen (14, 15).

Low immunogenicity is one of the challenges limiting the development of effective DNA vaccines (16, 17). In an effort to overcome this limitation, several research groups are focusing on DNA vaccine optimization and improved delivery methods, refining vaccine design through approaches including promoter design, codon optimization, adjuvants, electroporation use, prime/boost immunization, and “omics” (18, 19). Flagellin (FliC) is an adjuvant with proven potential for wide application. Flagellin-adjuvanted influenza vaccines have been tested in phase I and II clinical trials with acceptable safety and potent protective efficacy. In the clinical trials of VAX125, a fusion of flagellin (FljB) and the globular head of hemagglutinin was safe and able to induce greater HAI antibody levels and nearly complete seroprotection in subjects over 65 y old (20, 21). It is a dynamic structural protein of bacteria, and it contains four domains (D0, D1, D2, and D3) and is a TLR5 agonist that can induce a mixed Th1/Th2 immune response (22). FliCΔD2D3, which retains the highly conserved D0 and D1 regions of FliC but lacks the hypervariable regions D2 and D3, has immune adjuvant activity equivalent to that of FliC but has less antigenicity and triggers a more subdued systemic inflammatory response (23). These features prompted us to hypothesize that the flagellin variant FliCΔD2D3 could be used as a booster sequence for DNA vaccines.

In this study, the non-antibiotic-resistance-gene-containing vector *asd*-pVAX1 developed in the laboratory was used in the design of our candidate vaccine to generate a DNA vaccine with improved safety. The optimized *Salmonella typhimurium* flagellin adjuvant gene (*fliC*ΔD2D3) was fused with the candidate antigen S1 gene to enhance the immunogenicity of the vaccine. The humoral and cellular immune responses of the novel antibiotic-resistance gene-free DNA vaccine were evaluated in a mouse model.

## RESULTS

### Generation of DNA vaccine constructs

**FIG 1:**
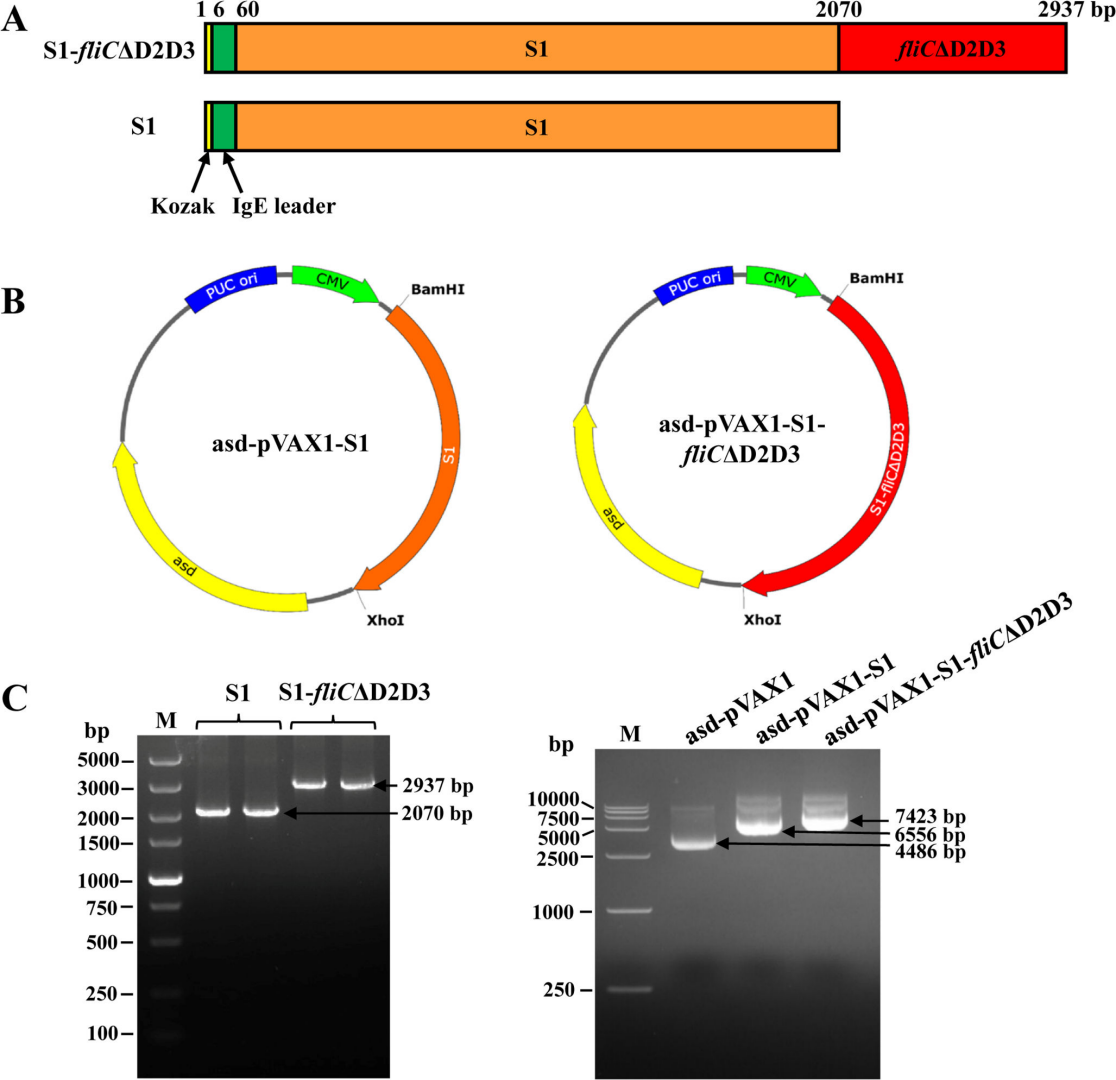
DNA vaccine design and construction. (**A**) Schematic diagram of two DNA vaccine constructs. Kozak (yellow) and IgE leader (green) sequences were included to promote protein expression. The S1 and *fliC*ΔD2D3 genes are indicated by orange and red colors, respectively. (**B**) Maps of the antibiotic-resistance gene-free DNA vaccines *asd*-pVAX1-S1 (left) and *asd*-pVAX1-S1-*fliC*ΔD2D3 (right). The kanamycin resistance gene of pVAX1 was replaced by the *asd* gene to create a DNA vaccine with improved safety. The S1 or *fliC*ΔD2D3 gene was individually inserted between *Bam*H I and *Xho* I by homologous recombination technology. (**C**) Agarose gel electrophoresis analysis of the S1 and S1-*fliC*ΔD2D3 genes of the DNA vaccine constructs (left) and the molecular weight of empty or recombinant DNA plasmids (right).

The amplified target genes S1 and S1-*fliC*ΔD2D3 containing an immunoglobulin (Ig) E leader peptide (Fig. 1A) were cloned into the antibiotic-resistance gene-free *asd*-pVAX1 vector, resulting in the generation of SARS-CoV-2 DNA vaccine constructs *asd*-pVAX1-S1 and *asd*-pVAX1-S1-*fliC*ΔD2D3, respectively (Fig. 1B). The successful construction of these recombinant plasmids was confirmed by PCR followed by electrophoresis of the products; two fragments could be detected at the expected molecular weights (Fig. 1C, left). The size and integrity of original *asd*-pVAX1 plasmids (~4.5 kb) or recombinant DNA vaccine constructs (~6.5 and ~7.4 kb) were identified (Fig. 1C, right). The results of the sequencing analysis further confirmed the insertion of the correct sequences in the desired orientation.

### *In vitro* confirmation of protein expression from the candidate vaccines

**FIG 2:**
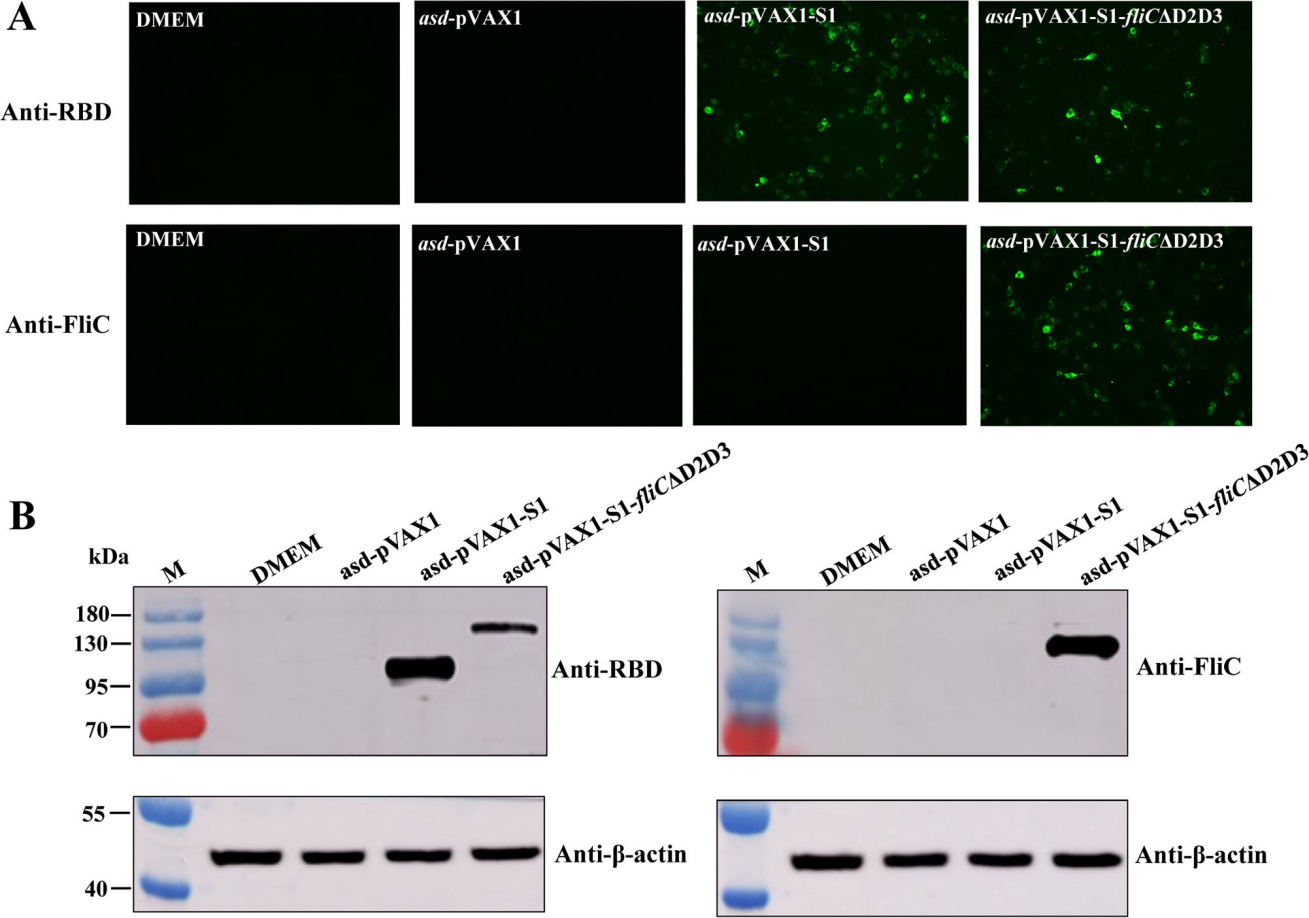
Expression verification of DNA vaccines in transfected cells. HEK293T cells were transfected with *asd*-pVAX1-S1, *asd*-pVAX1-S1-*fliC*ΔD2D3, or an empty vector (*asd*-pVAX1, used as a negative control). (**A**) Immunofluorescence analysis. Cells transfected with the indicated DNA plasmid were stained with antiRBD or antiFliC antibodies and then stained with an Alexa Fluor-488-labeled IgG secondary antibody (green). Images were captured using a fluorescence microscope at a magnification of 200×. (**B**) Western blot of lysates from cells transfected with the indicated DNA plasmid, probed with antiRBD (left) or antiFliC (right) antibodies and an antiβ-actin antibody as an internal control.

SARS-CoV-2 DNA vaccine candidates were evaluated for their ability to induce protein expression *in vitro* in HEK293T cells. The immunofluorescence assays were conducted to observe the intracellular expression of the target proteins in the transfected cells. The expression of S1 protein was successfully detected in cells transfected with *asd*-pVAX1-S1 or *asd*-pVAX1-S1-*fliC*ΔD2D3 by antiRBD antibodies, suggesting that the expressed proteins maintained their expected structural conformation. The S1-FliCΔD2D3 fusion protein expression was detected using antiFliC antibodies only in cells transfected with *asd*-pVAX1-S1-*fliC*ΔD2D3, i.e., not in cells transfected with *asd*-pVAX1-S1 or empty plasmid *asd*-pVAX1 (Fig. 2A).

Similarly, a western blot analysis confirmed that cells transfected with the recombinant constructs were able to express the S1 subunit and the S1-FliCΔD2D3 fusion protein after incubation with antiRBD antibodies (Fig. 2B, left). After incubation with antiFliC antibodies, S1-FliCΔD2D3 protein was detected only in the *asd*-pVAX1-S1-*fliC*ΔD2D3-transfected cells (Fig. 2B, right).

### The FliCΔD2D3-adjuvanted DNA vaccine induces enhanced humoral immune responses

**FIG 3:**
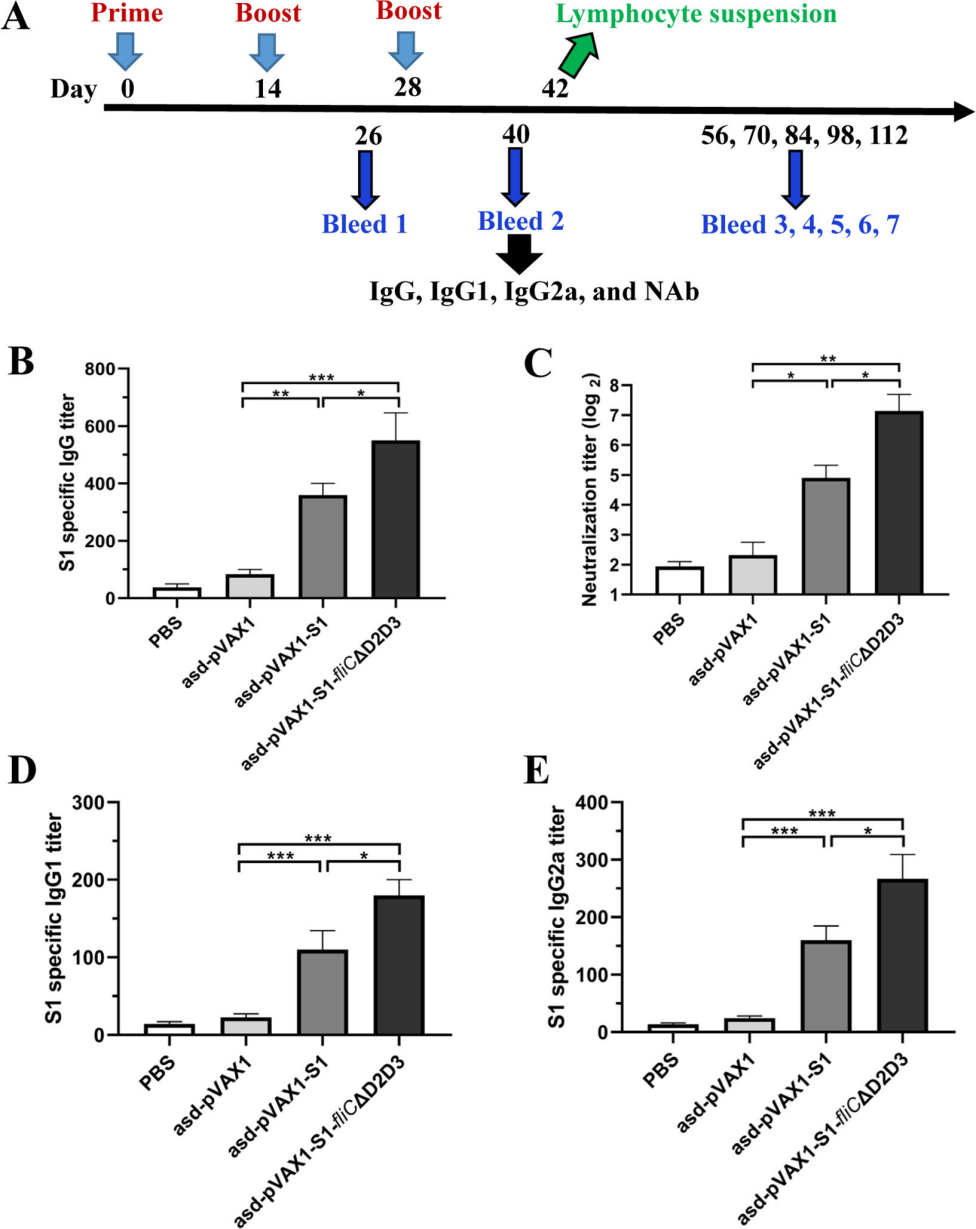
Specific antibody responses in mice after immunization with DNA vaccines. (**A**) Mouse immunization and sample collection schedule. Six- to eight-week-old female BALB/c mice (*n* = 9) were intramuscularly immunized with three doses of 100 µg of *asd*-pVAX1-S1, *asd*-pVAX1-S1-*fliC*ΔD2D3, empty vector, or PBS administered at 2-week intervals. Serum samples were collected at the indicated time points after immunization. (**B**) S1-specific IgG in the mouse sera on day 40, as determined by ELISA. (**C**) NAb against the SARS-CoV-2 pseudovirus as evaluated by a neutralization assay. SARS-CoV-2 pseudovirus-specific NAb titers were determined for sera samples collected on day 40 from mice immunized with the indicated vaccine candidate or control. S1-specific IgG1 titer (**D**) and IgG2a titer (**E**) on day 40, as evaluated by ELISA. *P < 0.05, **P < 0.01, and ***P < 0.001 were considered significant.

BALB/c mice were intramuscularly immunized with DNA vaccine candidates using a schedule of three injections administered at 2-week intervals. Two weeks after the last immunization, serum samples were collected to evaluate the levels of S1-specific IgG, IgG subtypes (IgG1 and IgG2a), and NAbs (Fig. 3A). The enzyme-linked immunosorbent assay (ELISA) data show that *asd*-pVAX1-S1 and *asd*-pVAX1-S1-*fliC*ΔD2D3 each induced significantly higher amounts of total S1-specific IgG against the SARS-CoV-2 S1 protein in the serum of immunized mice than phosphate buffered saline (PBS) or *asd*-pVAX1 (Fig. 3B). Moreover, the FliCΔD2D3-adjuvanted DNA vaccine induced potent NAb responses, which were significantly higher than those of sera from the *asd*-pVAX1- and *asd*-pVAX1-S1-immunized mice (Fig. 3C). Pseudovirus neutralization assays revealed that, compared with the empty vector, both S1 and S1-FliCΔD2D3 DNA vaccines elicited significantly higher titers of NAbs that prevent SARS-CoV-2 pseudovirus entry into HEK293/ACE2 cells. The S1-specific IgG, IgG1, and IgG2a titers in the sera from mice vaccinated with *asd*-pVAX1-S1-*fliC*ΔD2D3 were also significantly higher than those from mice vaccinated with *asd*-pVAX1-S1. Importantly, the analysis of IgG subclasses revealed comparable IgG1 and IgG2a titers (Fig. 3D and E), suggesting that Th1/Th2-mixed immune responses are induced by both designed DNA vaccine constructs.

### The FliCΔD2D3-adjuvanted DNA vaccine induces cytokine production following S1 stimulation in splenocytes isolated from vaccinated mice

**FIG 4:**
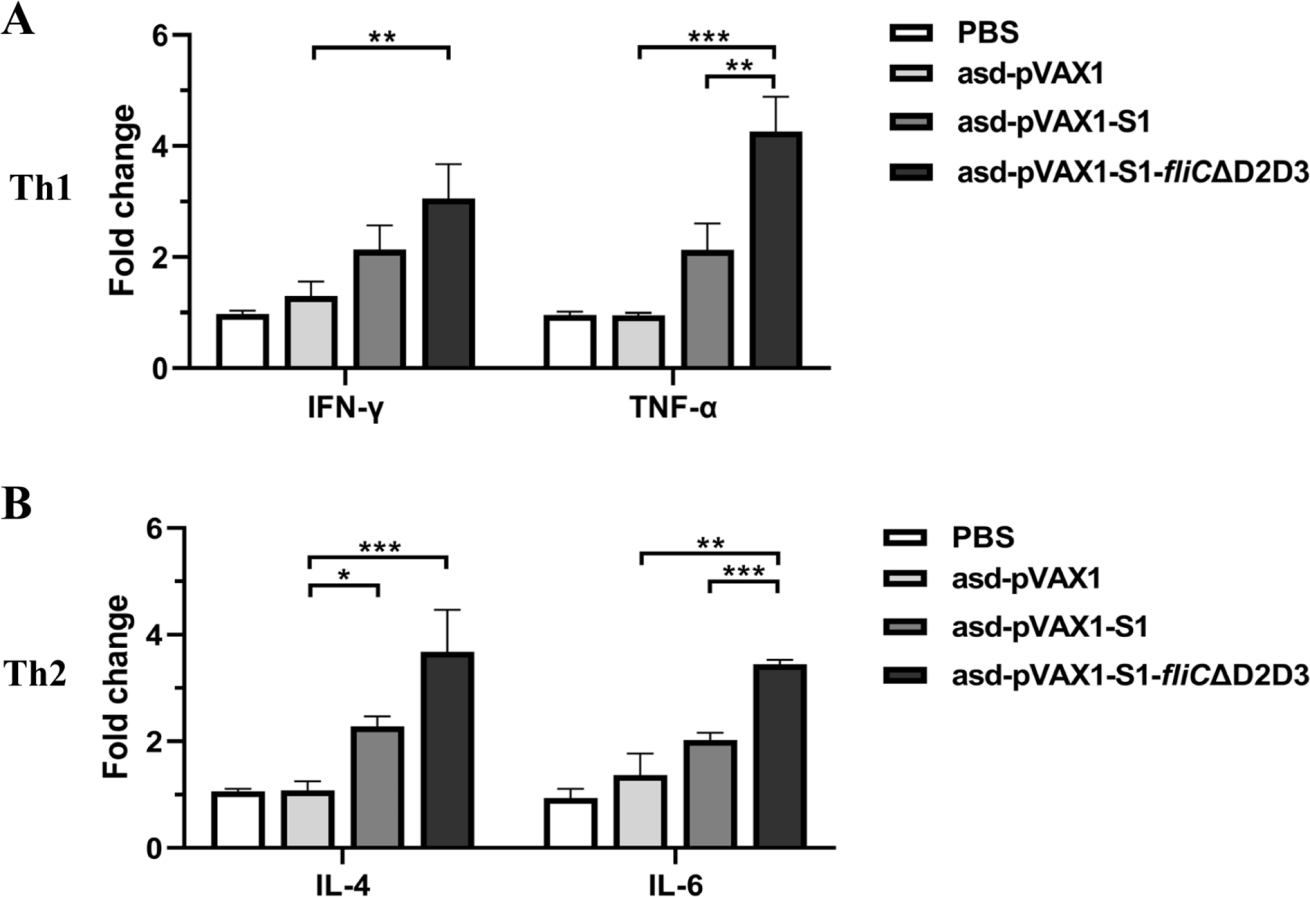
The cellular immune responses in mice after immunization with DNA vaccines. BALB/c mice were immunized via three intramuscular injections, administered at 2-week intervals, of 100 µg of vector, *asd*-pVAX1-S1, or *asd*-pVAX1-S1-*fliC*ΔD2D3. Splenocytes were collected at week 2 after the last vaccine dose, and, after their re-stimulation with recombinant SARS-CoV-2 S1 protein for 5 h, the mRNA levels of the Th1 cytokines IFN-γ and TNF-α (**A**) or the Th2 cytokines IL-4 and IL-6 (**B**) in these cells were evaluated. *P < 0.05, **P < 0.01, and ***P < 0.001 were considered significant.

The T-cell response against SARS-CoV-2 S antigen was evaluated by measuring the cytokine expression in splenic lymphocytes. Groups of vaccinated BALB/c mice were sacrificed at day 42 post-DNA vaccine administration. The splenocytes were harvested, and a single-cell suspension of the cells from each group was stimulated with the SARS-CoV-2 S1 protein for 24 h. In the *asd*-pVAX1-S1-*fliC*ΔD2D3 immunized group, the mRNA levels of the Th1-type cytokines IFN-γ (3.0-fold) and TNF-α (4.2-fold) were significantly increased compared with the *asd*-pVAX1 control group (Fig. 4A). Similarly, comparable levels of Th2-type cytokines (IL-4 and IL-6) were also significantly increased compared with the empty vector control (Fig. 4B). In addition, immunization with *asd*-pVAX1-S1-*fliC*ΔD2D3 induced significantly higher levels of TNF-α and IL-6 by splenocytes than immunization with *asd*-pVAX1-S1. These data suggest that the FliCΔD2D3-adjuvanted DNA vaccine induced enhanced immune responses that were Th1/Th2-mixed.

### The FliCΔD2D3-adjuvanted DNA vaccine induces long-term humoral immunity

**FIG 5:**
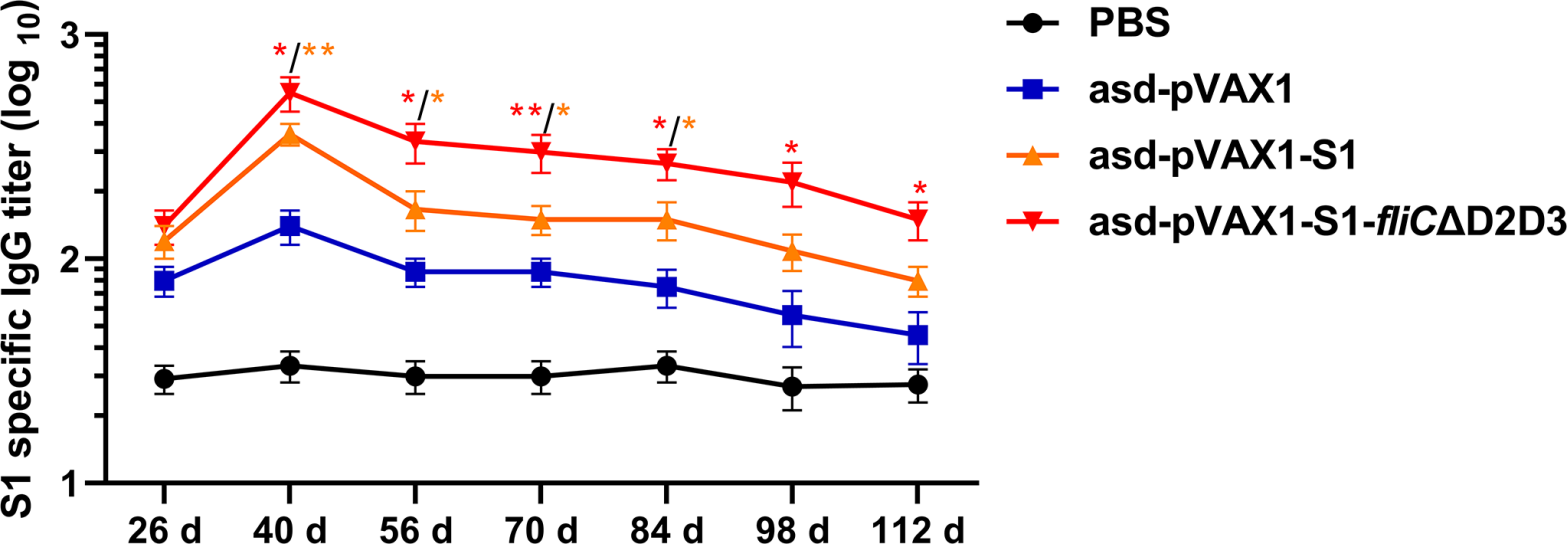
The longevity of IgG antibodies in the sera of mice following immunization with DNA vaccines. BALB/c mice (*n* = 6 per group) were intramuscularly injected three times, at 2-week intervals, with 100 µg of vector (*asd*-pVAX1), *asd*-pVAX1-S1, or *asd*-pVAX1-S1-*fliC*ΔD2D3. Serum samples were collected at the indicated time points after the first immunization. The titers of S1-specific IgG antibodies in these serum samples were evaluated by ELISA. The *asd*-pVAX1-S1-*fliC*ΔD2D3 group was statistically analyzed with the *asd*-pVAX1-S1 group, and the *asd*-pVAX1-S1 group was statistically analyzed with the empty vector control. *P < 0.05 and **P < 0.01 were considered significant.

To monitor the longevity of S1-specific IgG antibodies induced by the DNA vaccines in mice, blood samples were collected from the immunized animals over approximately 4 months (112 d) at 14-d intervals beginning after the second vaccine dose, and ELISAs were performed on the sera from these samples to determine the antibody titers. We analyzed the time course of S1-specific antibodies induced by the DNA vaccines. The IgG titers in the DNA vaccine groups reached their highest levels on day 12 post-final vaccine dose and then gradually decreased over the following 3 months. Vaccination with *asd*-pVAX1-S1-*fliC*ΔD2D3 induced higher IgG titers after the third vaccination than vaccination with *asd*-pVAX1-S1. Importantly, the S1-specific IgG titers remained high, even on day 112 (Fig. 5), suggesting that the DNA vaccine candidates generated an immune response in mice that was sustained for at least 4 months.

## DISCUSSION

DNA synthetic vaccine is a potential vaccine platform that could be used to prevent the transmission of diseases such as COVID-19. The design and synthesis of the DNA vaccine could be achieved quickly according to the sequence characteristics of the virus. However, a number of important questions regarding DNA vaccines have not yet been answered. For example, their potential for long-term protection and their safety profiles in large human populations remain unclear.

To further increase the efficacy of the DNA vaccine candidates, an IgE leader sequence was added in front of the sequence for the S1 protein. The N-terminal IgE leader peptide sequence could facilitate mRNA export and enhance protein expression. The IgE leader sequence was previously used in the INO-4800 DNA vaccine and in the MERS-CoV vaccine (24, 25). In addition, codon optimization of the S1 gene sequence may increase the immunogenicity of the vaccine. An optimized FliC adjuvant has been designed that can increase the immunogenicity of associated antigens (23). Furthermore, the pVAX1 vector applied here has been used in a number of other established DNA vaccines and has been proven to be safe for human use (26, 27). In the present study, our DNA vaccines were developed using an optimized antibiotic-resistance gene-free *asd*-pVAX1 vector. Two DNA vaccines, one containing only S1 (*asd*-pVAX1-S1) and the other containing S1 combined with a flagellin adjuvant (*asd*-pVAX1-S1-*fliC*ΔD2D3), were successfully constructed by non-antibiotic-resistance screening.

Immunofluorescence assays and western blot analyses showed the strong expression of these proteins in cells transfected with *asd*-pVAX1-S1 or *asd*-pVAX1-S1-*fliC*ΔD2D3. Meanwhile, the antiFliC antibody produced a strong signal only in the *asd*-pVAX1-S1-*fliC*ΔD2D3 transfected cells. These results demonstrate the ability of the antibiotic-resistance gene-free DNA vaccines to induce strong expression of the encoded proteins in mammalian cells and suggest that the expressed proteins have good immunoreactivity. Notably, the bands of the S1 and S1-FliCΔD2D3 proteins were larger than expected in the western blot assay. A similar finding has also been reported in full-length S-DNA vaccine studies (25); there are 22 potential N-terminal glycosylation points in the S protein (28), so the larger size may indicate that the proteins were glycosylated in the transfected cells.

The application of flagellin as an adjuvant is a promising approach that can enhance the antigenicity of immunogens. The latest research shows that flagellin could be administered with antigens of SARS-CoV-2 linked to the carboxyl and amino terminals of flagellin adjuvant derived from *Salmonella dublin* and antigens of Zika virus fused with *Salmonella typhimurium* flagellin as a novel mucosal adjuvant (29, 30), even in plant protein vaccines using self-replicating viral vectors (31). Several studies on DNA vaccines fused to full-length flagellin have been reported. Ajamian et al. found that the incorporation of HIV-1 gp41 into a FliC-based scaffold significantly augments gp41 immunogenicity and elicits modest membrane proximal external region-specific humoral responses in a mouse model (32). Ma et al. developed effective vaccines by fusing the Streptococcus equi antigen SeM with the FljB of *Salmonella abortus* (33). As expected, we found that the S1-specific IgG titers in the *asd*-pVAX1-S1-*fliC*ΔD2D3 group were likewise significantly higher than those of mice vaccinated with *asd*-pVAX1-S1. The inclusion of a DNA vaccine adjuvant can improve the quality of antibody responses, such as the level of NAb, owing to the ability of DNA vaccines to induce better antibody responses against conformational epitopes (34). Here, we reported serum NAb titers following DNA vaccination, which were determined by using a GenScript NAb detection kit. The measured NAb titer values demonstrate that the S1-FliCΔD2D3 DNA vaccine elicited significantly higher levels of NAb against the SARS-CoV-2 pseudovirus than S1 alone. The titer of NAb is comparable to that of the most advanced COVID-19 DNA vaccine, INO-4800, which encodes the full-length S protein (25). INO-4800 is currently being evaluated in clinical trials at several locations around the world (35). Considering the emergence of the COVID-19 VOC, Broderick et al. compared humoral and cellular immune responses against SARS-CoV-2 VOC in subjects immunized with the INO-4800 vaccine. The INO-4800 vaccination induced NAb against all variants tested. IFN-γ T-cell responses were fully maintained against multiple variants of concern (36, 37).

Cell-mediated immune responses also play a critical role in viral control, as SARS-CoV-2-specific cellular immune responses in the absence of a corresponding antibody response were found in individuals with a confirmed COVID-19 diagnosis who were asymptomatic or had mild symptoms (38). Noticeably, several clinical trials, including AG0302-COVID19, INO-4800, GX-19N, Covigenix VAX-001, COVID-eVax, and bacTRL-Spike, have been developed. These vaccines have stimulated both humoral and cellular immunity, except for GX-19N and AG0302-COVID19 (39). We here observed that the DNA vaccine is capable of inducing a cellular immune response complementary to the antibody response in a mouse model, as demonstrated by the observed cytokine expression from splenic lymphocytes. We found that *asd*-pVAX1-S1-*fliC*ΔD2D3 immunization induced strong Th1 and Th2 immune responses, evidenced by relatively high levels of IFN-γ, TNF-α, IL-4, and IL-6 expression in stimulated splenic lymphocytes, whereas *asd*-pVAX1-S1 immunization induced low levels of these cytokines. This is very important because successful DNA vaccination is known to induce both humoral and cellular responses in animals as well as in humans (40, 41). Furthermore, the use of FliC adjuvant immunization enhanced the induction of both Th1 and Th2 immune responses. The mixed Th1/Th2 responses induced by our COVID-19 DNA vaccine candidates suggest that these vaccines are unlikely to cause vaccine-associated enhanced respiratory disease, which is induced by Th2-biased vaccines (42).

Because SARS-CoV-2 infection induces relatively low levels of antibodies that decrease quickly and fail to provide long-term protection from reinfection, a successful COVID-19 vaccine will need to induce more persistent immune responses. Evaluation of our DNA vaccine candidates in mouse models revealed their ability to elicit immune responses against the SARS-CoV-2 S1 antigen. The primary antibody response in the serum started mounting 2 weeks after the second vaccine dose and reached its peak 2 weeks after the third dose. The serum levels of IgG against S1 antigen in mice were maintained even after 3 months post-final vaccine dose, suggesting that a long-term immune response is generated by the DNA vaccine candidate. This trend is in line with that of the DNA vaccine ZycoV-D, which is already approved for emergency use in India; when ZycoV-D was evaluated in mouse models, its specific antibody titer lasted 25 weeks (33, 43). In addition, *asd*-pVAX1-S1-*fliC*ΔD2D3 induced higher S1-specific IgG titers than *asd*-pVAX1-S1 over all assessed time points. This also indicates that *asd*-pVAX1-S1-*fliC*ΔD2D3 may induce strong memory immune responses following immunization again, similar to other DNA vaccine candidates that have been reported (44).

The mutations in the S protein may lead to a change in its conformation, which may change its antigenicity and then affect the design of the vaccine (45). The advantage of current DNA vaccine technology is the short time from design to clinical trial evaluation. Because a DNA synthetic vaccine can be designed and deployed quickly, it could be used as an effective countermeasure against SARS-CoV-2 VOCs. A wide range of strategies have been tested to improve the immunogenicity of DNA vaccines. An ideal DNA vaccine should have an optimized plasmid DNA construct and delivery system that yields a high protein expression level. In this study, plasmid optimization has shown the potential of DNA vaccines. Furthermore, other auxiliary methods, such as needle-free jet injection, microneedle delivery, and electroporation, represent alternative physical delivery mechanisms that could further enhance vaccine immunogenicity (25, 26). In addition, it has been proposed that the use of a bacterial delivery vector enhances the immune responses of the host against the viral infection (46, 47). More work is still needed to get the COVID-19 pandemic under control.

In summary, these results demonstrate the immunogenicity of the antibiotic-resistance gene-free DNA candidate vaccines *asd*-pVAX1-S1 and *asd*-pVAX1-S1-*fliC*ΔD2D3 in mice. This study provides crucial information regarding the selection of a safer DNA vector and an effective adjuvant for SARS-CoV-2 vaccine development. The FliCΔD2D3-adjuvanted S1 DNA vaccine is more potent at inducing both NAb and Th1/Th2 mixed immune responses than the DNA vaccine containing the S1 immunogen alone. This finding provides a new idea for the development of novel DNA vaccines against COVID-19 that could be further applied for the development of other vaccines.

## MATERIALS AND METHODS

### Mice and ethics statement

Female, 6–8-week-old BALB/c mice were purchased from Beijing Vital River Laboratory Animal Technology Co. Ltd. All mice were housed in isolators with controlled temperature, light, and ventilation. Pathogen-free water and food were supplied *ad libitum*. All animal studies were approved by the Committee on the Ethics of Animal Experiments of Yangzhou University [Approval ID: SYXK (Su) 2017-0044].

### Construction of DNA vaccine plasmids

The codon-optimized recombinant fusion gene sequence S1-*fliC*ΔD2D3, which is composed of the SARS-CoV-2 S1 sequence and the sequence of hypervariable region (D2 and D3)-deleted flagellin linked by flexible peptides (Gly_4_Ser)_2_, was commercially synthesized by GenScript Biotech Co., Ltd. (Nanjing, China). The sequences of SARS-CoV-2 S1 and *fliC* are based on Wuhan Hu-1 (GenBank accession no. MN908947.3) and *S. typhimurium* flagellin (GenBank accession no. CP001363.1), respectively. The S1 and S1-*fliC*ΔD2D3 gene sequences with Kozak and IgE leader sequences were amplified from the fusion gene template using the primers S1-F/S1-R and S1-F/S1-*fliC*ΔD2D3-R, respectively (Table 1). The IgE leader sequence is ATGGATTGGACTTGGATCTTATTTTTAGTTGCTGCTGCTACTAGAGTTCATTCT, as described previously (25). The resulting PCR products were individually subcloned into the antibiotic-resistance gene-free *asd*-pVAX1 plasmid DNA vector constructed previously (48) between *Bam*H I and *Xho* I by homologous recombination technology (Vazyme, Nanjing, China). Subsequently, the plasmid DNA constructs were separately transformed in *Escherichia coli* X6212 (*asd* ^−^) competent cells. After the heat-shock transformation step, the *E. coli* clones carrying the plasmid DNA constructs were isolated by plating the cells on a Luria-Bertani agar plate without diaminopimelic acid and antibiotics. Single clones were picked and used to extract plasmids for PCR identification. All plasmid sequences were confirmed by DNA sequencing (Tsingke, Beijing, China). The DNA vaccine plasmids (*asd*-pVAX1-S1 and *asd*-pVAX1-S1-*fliC*ΔD2D3) and empty vector (*asd*-pVAX1) were purified from *E. coli* using the endotoxin-free plasmid maxi kit (TIANGEN, Beijing, China) and used for transfection and animal studies.

**TABLE 1:** Sequences of primers used for the construction of DNA vaccine plasmids

Primer	Sequence (5′–3′)	Restricted site
S1-F	cttggtaccgagctc ***ggatcc*** gccaccatggattggacttg	*Bam*H I
S1-R	aacgggccctctaga ***ctcgag*** ttatcttgctcttctggggctgtt	*Xho* I
S1-*fliC*Δ-R	aacgggccctctaga ***ctcgag*** tcatctcagcaggctcaggac	*Xho* I

### *In vitro* expression analysis of the constructs by immunofluorescence

The *in vitro* expression of DNA vaccine candidates was checked by performing transfection experiments. HEK293T cells were seeded at a density of 2 × 10^5^ cells/well in 24-well plates and incubated at 37°C in a 5% CO_2_ incubator to be approximately 60%–80% confluent by the next day. The cells were then separately transfected with 0.5 µg of the *asd*-pVAX1-S1 or *asd*-pVAX1-S1-*fliC*ΔD2D3 DNA vaccine candidates or the control plasmid *asd*-pVAX1 using Opti-MEM medium (Gibco, Carlsbad, CA, USA) with Lipofectamine 3000 (Invitrogen, Carlsbad, CA, USA) in accordance with the manufacturer’s instructions and incubated at 37°C in a 5% CO_2_ incubator. After 5 h, the medium was replenished with complete DMEM containing 1% penicillin/streptomycin and 10% fetal bovine serum (Gibco). After 24 h, the cells were washed with PBS, fixed, and permeabilized by treatment with ice-cold methanol for 10 min. The cells were then washed twice and blocked with 5% bovine serum albumin (BSA)/PBS at room temperature for 2 h. After being washed, the cells were incubated with antiRBD rabbit polyclonal antibody (Sino Biological, Beijing, China) or antiFliC mouse antibody at a 1:1,000 dilution at 4°C overnight. The cells were then washed five times and stained with Alexa Fluor-488-labeled goat antirabbit or antimouse IgG secondary antibody (Abcam, Cambridge, MA, USA) at a 1:1,000 dilution at 37°C for 2 h. The cells were washed three times with PBS and mounted with 20% glycerol. Stained cells were visualized under a Leica fluorescence microscope.

### *In vitro* expression analysis of the constructs by western blot

HEK293T cells (60%–80% confluent) in 24-well plates were transiently transfected with 1.0 µg of *asd*-pVAX1-S1, *asd*-pVAX1-S1-*fliC*ΔD2D3, or control plasmid *asd*-pVAX1 using Lipofectamine 3000 (Invitrogen) in accordance with the manufacturer’s instructions, followed by incubation at 37°C in a 5% CO_2_ incubator. At 24 h post-transfection, the medium was removed, and the cells were washed with PBS and lysed with cell lysate buffer (Beyotime, Shanghai, China) for analysis by western blot.

The lysates were harvested and subjected to electrophoresis on a 12% SDS-PAGE gel, and the separated proteins were transferred to nitrocellulose membranes. These membranes were blocked with tris-buffered saline with 0.05% Tween 20 (TBST) containing 2% BSA for 2 h at room temperature. After being washed, the membranes were incubated with rabbit antiRBD polyclonal antibody (Sino Biological) or mouse antiFliC antibody at a 1:1,000 dilution at 4°C overnight. After being washed repeatedly, the membranes were incubated with horseradish peroxidase (HRP)-conjugated goat antirabbit IgG or antimouse IgG (1:5,000) at 37°C for 1 h. The protein blots were examined using ECL reagents (Vazyme), and images of the blots were captured using an Amersham Imager 600 imaging system (GE Healthcare, Piscataway, NJ, USA). Additionally, β-actin was detected with an antiβ-actin antibody (Sigma, MO, USA) at a 1:2,000 dilution as a loading control.

### Animal immunization and sample collection

The immunogenicity study for the antibiotic-resistance gene-free DNA vaccine was carried out in BALB/c mice. Female 6–8-week-old BALB/c mice were randomly divided into four experimental groups (nine mice/group) and immunized intramuscularly three times, each with a dose of 100 µg/100 µL *asd*-pVAX1-S1 or *asd*-pVAX1-S1-*fliC*ΔD2D3 DNA vaccine candidates, on days 0, 14, and 28, respectively. Mice injected with the empty plasmid *asd*-pVAX1 or PBS served as the vehicle or negative control group, respectively. Serum samples were collected from animals on day 26 and on days 40, 56, 70, 84, 98, and 112 (2-week intervals) for antibody response detection. At 2 weeks after the last vaccination, the spleens of immunized mice were collected to assess the cellular immune responses.

### Enzyme-linked immunosorbent assay

The titers of S1-specific IgG, IgG1, and IgG2a in serum from immunized mice were determined by indirect ELISA, as previously described (49). Briefly, 96-well plates were coated with 0.5 µg/mL SARS-CoV-2 S1 protein (Sino Biological) at 4°C overnight. The plates were washed three times with PBS containing 0.1% Tween-20 (PBST) before being blocked with 1% BSA in PBST for 2 h at 37°C. After the plates were washed again, twofold serial dilutions of mouse serum, starting at 1:50, were added to the wells and incubated for 2 h at 37°C. HRP-conjugated goat antimouse IgG (1:10,000), IgG1 (1:5,000), or IgG2a (1:5,000) secondary antibodies (Abcam) were then added at the recommended concentrations and incubated for 1 h at 37°C. After the plates were extensively washed, tetramethylbenzidine substrate was added to induce a colorimetric reaction. The reaction was stopped with 2 M H_2_SO_4_, and the absorbance at 450 nm was read on a microplate reader (BioTek, Winooski, VT, USA). The cutoff value was defined as the mean plus two standard deviations of the negative control value. The antibody titers were defined as the reciprocal of the highest dilution of samples that had a reading above the cutoff value.

### Pseudoviral neutralization assay

The NAb titers in serum samples from vaccinated mice were detected using the SARS-CoV-2 Pseudovirus Neutralization Kit_Luc Reporter (GenScript, Nanjing, China) in accordance with the manufacturer’s instructions. Briefly, the heat-inactivated serum samples were serially diluted fivefold with a starting dilution of 1:5 in 25 µL of volume. After an equal volume of SARS-CoV-2 pseudovirus was added, they were incubated for 1 h at room temperature. Then, 50 µL of the serum/virus mixture was added to the 96-well plates that had been preseeded with 50 µL of HEK293/ACE2 cells per well. After the plates were incubated for 24 h at 37°C with 5% CO_2_, 50 µL/well completed DMEM was added. At 24 h after infection, the cells were washed with PBS and then treated with 50 µL/well of Luciferase substrate (Promega, Madison, WI, USA). The luciferase activities were measured using a microplate reader (BioTek). The NAb titers were defined as the reciprocal serum dilution at which the amount of RLU was reduced by 50% compared with the amount of RLU in virus-control wells after subtraction of the amount of background RLU in cell-control wells.

### Isolation of splenocytes from immunized mice

Two weeks after the last vaccination, spleens were collected from the mice to assess the cellular immune responses. Splenocytes were prepared from immunized mice as described previously (49). Single lymphocyte suspensions were seeded into 24-well plates (1 × 10^6^ cells/well in 0.5 mL) in complete RPMI 1640 containing 10% fetal bovine serum and 1% penicillin-streptomycin L-glutamine (Gibco). The cells were stimulated with 5 µg/mL SARS-CoV-2 S1 protein (Sino Biological). After 5 h, the cells were collected separately for RNA extraction and reverse transcription (RT)-PCR quantification.

### RNA extraction and RT-PCR quantification of cytokines

Total mRNA of the splenic lymphocytes from immunized mice was obtained using a total RNeasy Plus Mini kit (Qiagen, Hilden, Germany), and cDNA was synthesized from the mRNA using a PrimeScrip RT reagent kit (Takara, Dalian, China) in accordance with the manufacturer’s instructions. The mRNA levels of the cytokines IFN-γ, IL-4, TNF-α, and IL-6 were detected by qRT-PCR analysis using SYBR Green master mix (Roche Diagnostics, Tokyo, Japan) on an ABI 7500 Fast Real-Time PCR System. PCR amplification was performed in a total volume of 20 µL containing 10 µL of 2× SYBR Premix Ex Taq II, 2 µL of diluted cDNA, and 0.8 µL of each primer. The real-time PCR program started with a denaturing step at 95°C for 30 s, followed by 40 cycles of 95°C for 5 s and 60°C for 60 s. Relative quantifications of the mRNA of target genes are shown as the comparative threshold cycle number for each sample (2^−ΔΔCT^). Gene expression was compared with the corresponding β-actin level. The primers used for qRT-PCR were synthesized by GenScript Biotech Co., Ltd., and their sequences are shown in Table 2.

**TABLE 2:** Sequences of primers used for quantitative real-time PCR

Gene	Primer sequences (5′–3′)	Product size (bp)	Accession no.
IFN-γ	F: actggcaaaaggatggtgac	237	NM_008337.4
	R: tgagctcattgaatgcttgg		
IL-4	F: tcaacccccagctagttgtc	177	NM_021283.2
	R: tgttcttcgttgctgtgagg		
TNF-α	F: agcccccagtctgtatcctt	212	NM_013693.3
	R: ctccctttgcagaactcagg		
IL-6	F: agttgccttcttgggactga	159	NM_031168.2
	R: tccacgatttcccagagaac		
β-Actin	F: agccatgtacgtagccatcc	228	NM_007393.5
	R: ctctcagctgtggtggtgaa		

### Statistical analysis

All data are expressed as the mean ± standard error of the mean. The statistical analyses were performed using GraphPad 8.0 software, and the data were analyzed using Student’s *t*-tests and one-way analyses of variance. Differences with *P < 0.05, **P < 0.01, and ***P < 0.001 were considered statistically significant.
